# Isolation and ex vivo cultivation of single myofibers from porcine muscle

**DOI:** 10.1007/s11626-020-00492-z

**Published:** 2020-09-22

**Authors:** Katja Stange, Hellen Elisa Ahrens, Julia von Maltzahn, Monika Röntgen

**Affiliations:** 1 Institute of Muscle Biology and Growth, Growth and Development Unit, Leibniz Institute for Farm Animal Biology (FBN), Wilhelm-Stahl-Allee 2, 18196 Dummerstorf, Germany; 2 Research Group Stem Cells in Regeneration of Skeletal Muscle, Leibniz Institute on Aging, 07745 Jena, Germany

**Keywords:** Porcine muscle fiber, Myofiber isolation, Nuclear density, Skeletal muscle, Pig

## Abstract

The isolation and cultivation of intact, single myofibers presents a superior approach for studying myogenic cells in their native position. The cells’ characteristics remain more similar to muscle tissue than in cell culture. Nevertheless, no routinely used method in higher vertebrates exists. Therefore, we aimed at establishing the isolation and cultivation of single myofibers from porcine muscle. For the first time, we implemented the isolation of intact myofibers from porcine fibularis tertius muscle by enzymatic digestion and their subsequent cultivation under floating conditions. Confocal microscopy showed intact myofibrill structures in isolated myofibers. Myogenic cells were able to proliferate at their parent myofiber as shown by the increase of myonuclear number during culture. Additionally, the described method can be used to investigate myogenic cells migrated from isolated myofibers. These cells expressed myogenic markers and were able to differentiate. In the future, our method can be used for genetic manipulation of cells at myofibers, investigation of growth factors or pharmacological substances, and determination of interactions between myofibers and associated cells. Working with isolated myofibers has the potential to bridge conventional cell culture and animal experiments. Adapting the method to porcine muscle allows for application possibilities in veterinary medicine as well as in biomedical research, which cannot be addressed in rodent model systems.

## Introduction

Studying skeletal muscle functionality in vitro is of utmost importance in order to elucidate cellular and molecular mechanisms controlling muscle development, maintenance, and metabolism, which also play a role in disease pathologies or tissue repair (Shefer and Yablonka-Reuveni 2005; Komiya *et al.* 2015; Stuelsatz *et al.* 2017). Myogenic stem cells, called satellite cells, were originally named by their localization between sarcolemma and basal lamina within their niche and are the main source for myonuclei (Mauro 1961). The myogenic cell population, including satellite cells and their progeny, extensively proliferates during early postnatal development and undergoes myogenic differentiation to enable muscle fiber growth (Swatland 1977; Campion *et al.* 1981; Miersch *et al.* 2017). Later on, adult satellite cells become quiescent and preserve the stem cell population (Mesires and Doumit 2002).

A broad spectrum of sophisticated methodological approaches is a prerequisite for investigating these processes. Working with tissue sections or homogenates can provide valuable information, but is restricted to end point analyses and does not enable dynamic investigations of tissue or specific cell populations. In vitro culture systems on the other hand allow investigating dynamic processes over time and under defined conditions, i.e., siRNA-mediated knockdown, application of growth factors, or conditioned media. To study the function of myogenic cells in vitro, two different approaches exist: First, myogenic cells can be isolated to obtain primary cell cultures. This involves mincing and digestion of the tissue to dissociate myogenic cells from their physiological environment (Rosenblatt *et al.* 1995; Stuelsatz *et al.* 2017). Thereby, a very heterogeneous mixture of different cells is liberated and their separation as well as the discrimination of cellular subpopulations requires additional purification steps.

Second, intact, single myofibers can be isolated while myogenic cells remain in their endogenous niche (Yablonka-Reuveni and Rivera 1994; Zammit *et al.* 2004; Stuelsatz *et al.* 2017). Working with isolated muscle fibers allows investigating the interrelation of myogenic cells with their parent myofiber (Shefer and Yablonka-Reuveni 2005; Stuelsatz *et al.* 2017; Huttner *et al.* 2019). Myofibers can either be cultivated in suspension (floating culture), where myogenic cells will remain at their in situ position on the fiber. By this means, the cells’ microenvironment within the myofiber is maintained and naturally occurring processes like cell activation, proliferation, cluster formation, and differentiation can be mimicked ex vivo (Rosenblatt *et al.* 1995; Shefer and Yablonka-Reuveni 2005; Pasut *et al.* 2013; Gallot *et al.* 2016). For instance, it was shown that the Myosin heavy chain expression pattern of isolated fibers is more similar to muscle tissue than that of myotubes grown in primary cell cultures (Komiya *et al.* 2015). Hüttner and colleagues also concluded that the isolation and cultivation of single myofibers in suspension culture is superior in assessing stem cell functionality compared with cell culture systems (Huttner *et al.* 2019). The myofiber culture system also offers the possibility to conduct knockdown and overexpression studies aiming for detailed information on specific targets or to test for the effect of (pharmacological/toxic) substances (Shefer and Yablonka-Reuveni 2005; Anderson *et al.* 2012; Keire *et al.* 2013; Gallot *et al.* 2016; Ahrens *et al.* 2019). In addition, a variety of genetically modified reporter mouse lines are available, e.g., Pax7-GFP (Sambasivan *et al.* 2009) Myf5-YFP mice (Kuang *et al.* 2007). Isolated fibers can also be plated on coated dishes in order to promote cell adhesion. Cells will then migrate from their parent myofiber and attach to the surface of the cell culture dish before undergoing myogenic proliferation and differentiation (Beauchamp *et al.* 2000; Stuelsatz *et al.* 2017). Compared with conventional cell isolation by tissue dissociation, myogenic cultures obtained from single myofibers will have the advantage to be of higher purity (Rosenblatt *et al.* 1995; Etienne *et al.* 2020).

The isolation of single myofibers was first described by Bekoff and Betz (1977a,b) from the *flexor digitorum brevis* muscle (FDB) in adult rats. The protocol was later on modified by Bischoff (1986). In the following decades, many others optimized the method and adapted it to other rodent muscles. Today, single myofiber isolation is optimized very well in mice and rats of different strains and ages for a series of muscles including *extensor digitorum longus* (EDL), *tibialis anterior* (TA), and FDB, as well as soleus, plantaris, diaphragm, masseter, and extraocular muscles (Rosenblatt *et al.* 1995; Shefer and Yablonka-Reuveni 2005; Pasut *et al.* 2013; Gallot *et al.* 2016; Sawano *et al.* 2016; Stuelsatz *et al.* 2017; Huttner *et al.* 2019). Muscles in these extensively studied model organisms are of course quite small, making them easier to handle but also yielding only a limited amount of myofibers (Rosenblatt *et al.* 1995).

The transferability of results obtained in cell lines, non-vertebrates, or rodents cannot be assumed automatically for human research. The pig is widely accepted as an excellent model system for humans since among others organ size, eating habits, and metabolism are relatively similar (Fu *et al.* 2013; Ferenc *et al.* 2014). In addition, the pig is often used as a model system in preclinical studies and medical tests (Ferenc *et al.* 2014). In contrast to mouse models, genetically modified reporter lines are not available for pigs and their development, maintenance, and use would be more biologically complicated, expensive, and time-consuming and are therefore not feasible for a large number of target genes. However, most pigs are farm animals, raised and used for meat production. Therefore, the majority of porcine studies, e.g., regarding positive modulation of muscle growth, are in vivo animal experiments, which we should aim to reduce wherever possible. Unfortunately, for large mammals, like the pig, working with isolated and cultivated myofibers or cells derived from these fibers is still at a very early stage and not routinely used.

Functional investigations of myogenic cells within their niche are not possible with conventional cell culture approaches making the method of myofiber isolation and cultivation indispensable (Rosenblatt *et al.* 1995; Pasut *et al.* 2013). Establishing the isolation and cultivation of single, intact myofibers would be essential to complement the methodological spectrum within the porcine model system.

## Material and Methods

### Isolation of single myofibers from porcine FT muscle

Here, we present the isolation and cultivation of single myofibers from porcine *Musculus fibularis tertius* (FT) for the first time. We decided to use the FT muscle, since its size, anatomy, and accessibility make it very suitable for myofiber isolation. The location of the FT muscle at the anterior site of the lower hind leg is comparable with the EDL or TA muscle in rodents, which are routinely used for myofiber isolation. The FT muscle (König and Bragulla 2009), also named *peroneus tertius* or *anterior fibularis*, was first identified as a part of the EDL muscle and later on named as a separate entity (Yammine and Eric 2017). The muscle is present in the majority of humans (93%) (Yammine and Eric 2017) and also found in other species, including horse, ruminants, and pigs, but is missing in carnivores or rabbits (Denoix 2010). While the muscle is fibrous in horse, the muscle body is prominent in ruminants and pigs being responsible for tarsus flexion (Denoix 2010).

**Figure 1.**
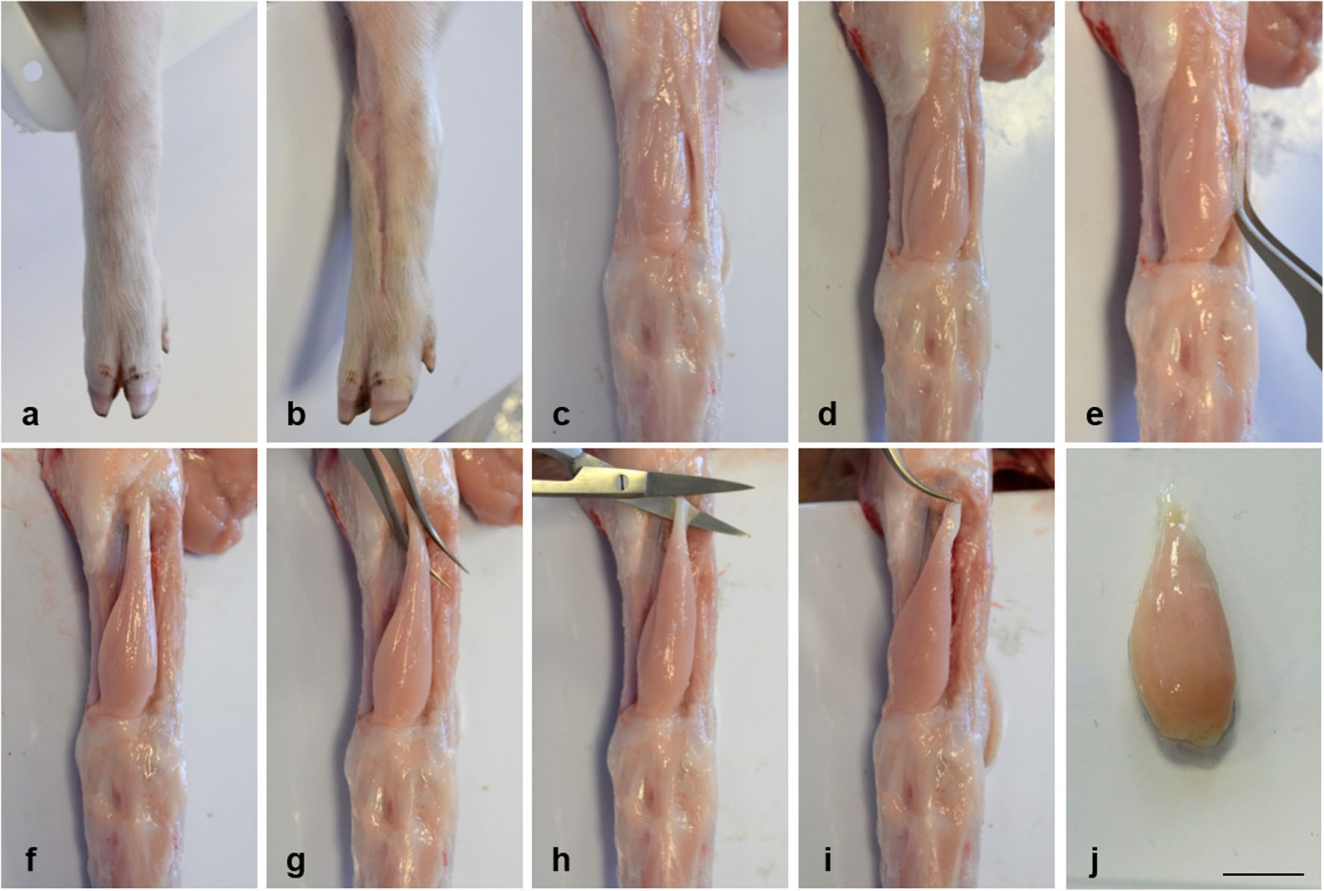
Muscle dissection from pig hind limb. After cleaning the hind limb with ethanol (*a*), the skin was cut (*b*) and removed carefully using a scalpel until the underlying tissue can be seen (*c*). Visible adipose and connective tissue was removed using fine, sharp scissors (straight or curved), Vannas spring scissors, and curved forceps (*d*, *e*) to expose the *Musculus fibularis tertius* (*f*). The *M*. *fibularis tertius* was carefully separated from the fibula, and the surrounding tissue and curved forceps were used to expose the proximal tendon (*g*) which is cut with fine scissors (*h*). The proximal tendon was carefully fixed (*i*) and the distal tendon was cut with a scalpel in order to retrieve the whole muscle (*j*). The muscle should be handled only at the tendons without stretching or touching the muscle body which is crucial to avoid damaging the fibers (Rosenblatt *et al.* 1995). All instruments used for muscle dissection were obtained from Fine Science Tools (FST) and cleaned with 70% ethanol. *Scale bar* represents 1 cm.

Four-day-old, female German Landrace piglets from the experimental pig unit of the Leibniz Institute for Farm Animal Biology were kept and sacrificed following the guidelines set by the Animal Care Committee of the State Mecklenburg-Western Pomerania, Germany, based on the German Law of Animal Protection. The FT muscle was removed as shown in Fig. 1. The whole muscle was incubated at 37°C in 5 ml digestion solution composed of DMEM with 4.5 g/l glucose (PAN-Biotech, Aidenbach, Germany), 0.2% collagenase type I (*Clostridium histolyticum*, Merck, Darmstadt, Germany), and 0.1% elastase from porcine pancreas (SERVA, Heidelberg, Germany). The tissue was digested for 7 h and shaken about every 30 min. After 4 h of digestion, 0.2% collagenase type I (500 μl) was freshly added.

The majority of previously described protocols used collagenase type I for tissue digestion with concentrations ranging from 0.2 to 0.4% (Bekoff and Betz 1977a,b; Shefer and Yablonka-Reuveni 2005; Wilschut *et al.* 2010b; Pasut *et al.* 2013; Stuelsatz *et al.* 2017; Huttner *et al.* 2019). The aforementioned protocols describe a digestion time of 30 min up to 3 h depending on the selected muscle type, species, and age of the animal. The separation of single myofibers from the muscle body is macroscopically visible, and we found that approximately 7 h of digestion were necessary for FT muscle of 4-d-old piglets. Because of this long time span, we decided to add fresh collagenase in the meantime. Even for rodent muscles, it was described that within thick muscles, single fibers can be hard to free because connective tissue is not digested sufficiently by collagenase alone (Rosenblatt *et al.* 1995). Thus, some protocols used additional digestion enzymes, namely elastase, protease, and dispase for myofiber isolation in rodents (Komiya *et al.* 2015; Sawano *et al.* 2016). We also found that connective tissue in porcine FT could not be digested by collagenase alone even after prolongation of the digestion time. Besides collagens as the main fibrous component of extracellular connective tissue, elastin is frequently found in elastic fibers of connective tissue (Worthington *et al.* 2016). Porcine elastase is much more potent than human elastase and therefore suitable to digest extensive fiber networks (Worthington *et al.* 2016). Therefore, we used 0.1% porcine elastase in addition to 0.2% collagenase to successfully isolate myofibers.

The digested muscle was transferred into a non-coated 6-well plate (Falcon, Corning, Wiesbaden, Germany) generously filled with DPBS (PAN-Biotech, Aidenbach, Germany). The muscle was pipetted up and down rigorously by using a trimmed, 5-ml pipette tip coated with horse serum (HS, Sigma-Aldrich, Hamburg, Germany). Previous coating or rinsing of all materials with HS shall prevent sticking of the fibers to the surface (Rosenblatt *et al.* 1995; Gallot *et al.* 2016). Next, the tissue was placed into a new well of the plate, again generously covered with DPBS and pipetted up and down. The procedure was repeated 3 to 4 times until single myofibers start to dissociate from the remaining muscle tissue.

### Isolated single myofibers in suspension culture

Twenty to 30 single myofibers were transferred into a 4-well plate equipped with a HS-coated cover glass using a HS-coated glass Pasteur pipette. Cultivation medium (DMEM with 4.5 g/l glucose (PAN-Biotech, Aidenbach, Germany) containing 10% HS, 0.5% chicken embryo extract (Biomol, Hamburg, Germany), 100 U/ml penicillin/streptomycin (PAN-Biotech), and 2.5 μg/ml amphotericin B (PAN-Biotech)) was carefully added and myofibers were cultured in a humidified atmosphere containing 5% CO_2_ at 37°C.

**Figure 2.**
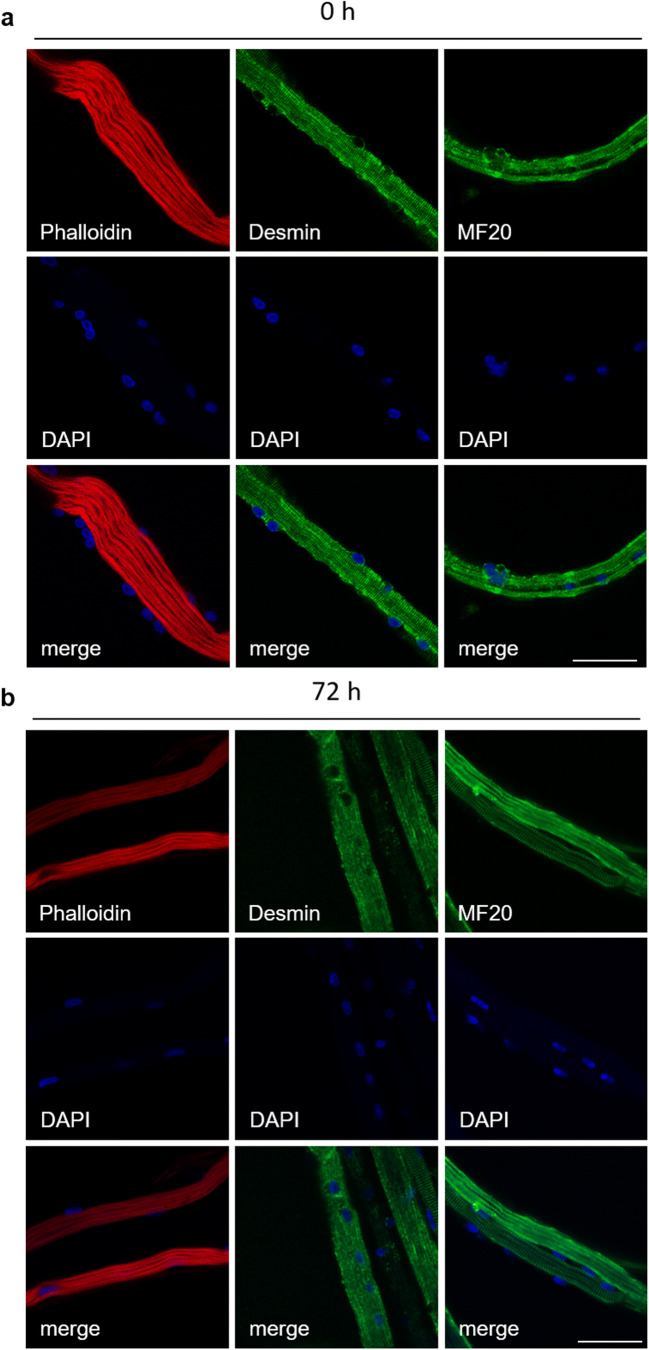
Analysis of freshly isolated and cultivated myofibers for F-Actin, Desmin, and Myosin. Single myofibers were stained directly after isolation (0 h, *a*) or after 72 h in suspension culture (*b*) before staining. Myofiber structure was visualized via LSM800 (Zeiss) and the corresponding ZEN software by using a fluorescent Phalloidin conjugate (F-Actin) or specific antibodies against Desmin or Myosin (MF20). Cell nuclei were stained using DAPI. *Scale bar* represents 50 μm.

Expression of myogenic markers and structural integrity of the isolated myofibers are a prerequisite for the applicability of the method. Directly after isolation (Fig. 2*a*) and after 72 h of cultivation in suspension culture (Fig. 2*b*), myofibers were stained against the myogenic filament proteins F-Actin, Desmin, and Myosin. Single myofibers were fixed with 2% PFA in PBS and washed with DPBS. To visualize F-Actin, myofibers were incubated with Phalloidin CruzFluor 594 Conjugate (Santa Cruz Biotechnology, Heidelberg, Germany) for 1 h, washed with DPBS, and mounted with ROTI Mount FluorCare DAPI (Carl Roth, Karlsruhe, Germany). To visualize Desmin or Myosin, immunostaining was performed. Myofibers were permeabilized with 0.1% Triton X-100 in PBS for 15 min and blocked in PBS containing 5% HS and 0.1% Triton X-100 for at least 1 h. Subsequently, myofibers were incubated with primary antibodies overnight: mouse anti-Desmin (clone D-33, DAKO, Hamburg, Germany, 1:80 in PBS containing 5% HS and 0.1% Triton X-100) or mouse anti-MHC (MF20, undiluted supernatant, Developmental Studies Hybridoma Bank). Samples were washed with DPBS and incubated for 45 min with a rabbit anti-mouse IgG (H+L) secondary antibody conjugated to Alexa Fluor^®^ 488 (1:1000, Thermo Fisher Scientific Waltham, MA). Myofibers were mounted with ROTI Mount FluorCare DAPI (Carl Roth). Each myofiber contains several myofibrils with their functional units named sarcomeres (reviewed in Mukund and Subramaniam 2020). Fibrous Actin (F-Actin, here visualized by a Phalloidin conjugate) is a thin protein filament of the myofibrils, whereas Myosin isoforms are found in the thick filaments. The Z-disk is present at the end of each sarcomere holding the thin filaments together. Z-disk-associated proteins, e.g., Desmin, also connect adjacent myofibrils to each other. The anti-parallel orientation of Desmin expression can be seen in Fig. 2. The structure of single myofibers remained intact after isolation as well as during the cultivation period. Adjacent nuclei can be seen within each myofiber, also after 3 d of cultivation, showing that our isolation and cultivation method is suitable to remain the cells’ position on the fiber.

## Results and Discussion

### Nuclear density

Myonuclei should be found at their parent myofiber throughout cultivation without being liberated. The myonuclear number was determined as a measure and showed that the cells remained at the isolated fibers and were even able to proliferate. Single myofibers were fixed immediately after isolation or after 4 d of cultivation with 2% paraformaldehyde (PFA) in PBS, washed with DPBS, and mounted with ROTI Mount FluorCare DAPI (Carl Roth). Microscopic images were recorded using Leica DM4000B and the corresponding software Leica QWin V3 (Wetzlar, Germany), and the nuclear density (number of nuclei per mm fiber) was determined using Fiji (ImageJ 2.0.0-rc 69/1.52p). For each animal, an average of 16 images and 15,267-μm fiber were quantified for each sample. The SigmaPlot 13.0 (Systat Software Inc. San Jose, CA) software was used for statistical analyses. One-tailed *t* test was performed after normality test (Shapiro-Wilk) and equal variance test (Brown-Forsythe) were passed. Directly after isolation, 34.41 ± 7.17 nuclei/mm were found at myofibers, whereas this value enhanced significantly to 41.66 ± 7.73 nuclei/mm within 4 d of cultivation. This shows that nuclei remain at the parent myofiber under the selected cultivation conditions instead of migrating to the surface of the culture dish. Furthermore, cells are also able to proliferate at isolated myofibers. To our knowledge, there are no such data available for porcine-isolated myofibers, yet. The nuclear density significantly changes during growth (Wada *et al.* 2003; Bachman *et al.* 2018), and data from the early postnatal growth phase as investigated here are quite rare. Myofibers of TA muscle isolated from newborn mice showed a myonuclear number just below 30 myonuclei/mm fiber (Wada *et al.* 2003) being comparable with 4-d-old piglets. For myofibers isolated from 7- to 15-wk-old mice, a nuclear density of 30–57 nuclei/mm fiber in EDL and 35–77 nuclei/mm fiber in soleus muscle was reported (Bruusgaard *et al.* 2003). In the *anterior latissimus dorsi* (ALD) muscle of adult chicken, the nuclear density was comparable (38 ± 2 nuclei/mm) (McCormick and Schultz 1994). These data are also in accord with the nuclear density reported here. In mice, the myonuclear number increased 2.8-fold during the first 5 wk of life (Wada *et al.* 2003). Therefore, assuming that the number of nuclei increases during fiber growth/hypertrophy in order to keep the nuclear domain constant (Bruusgaard *et al.* 2003), the nuclear density could be considerably higher in myofibers isolated from older pigs.

### Obtaining myogenic cells from isolated myofibers

**Figure 3.**
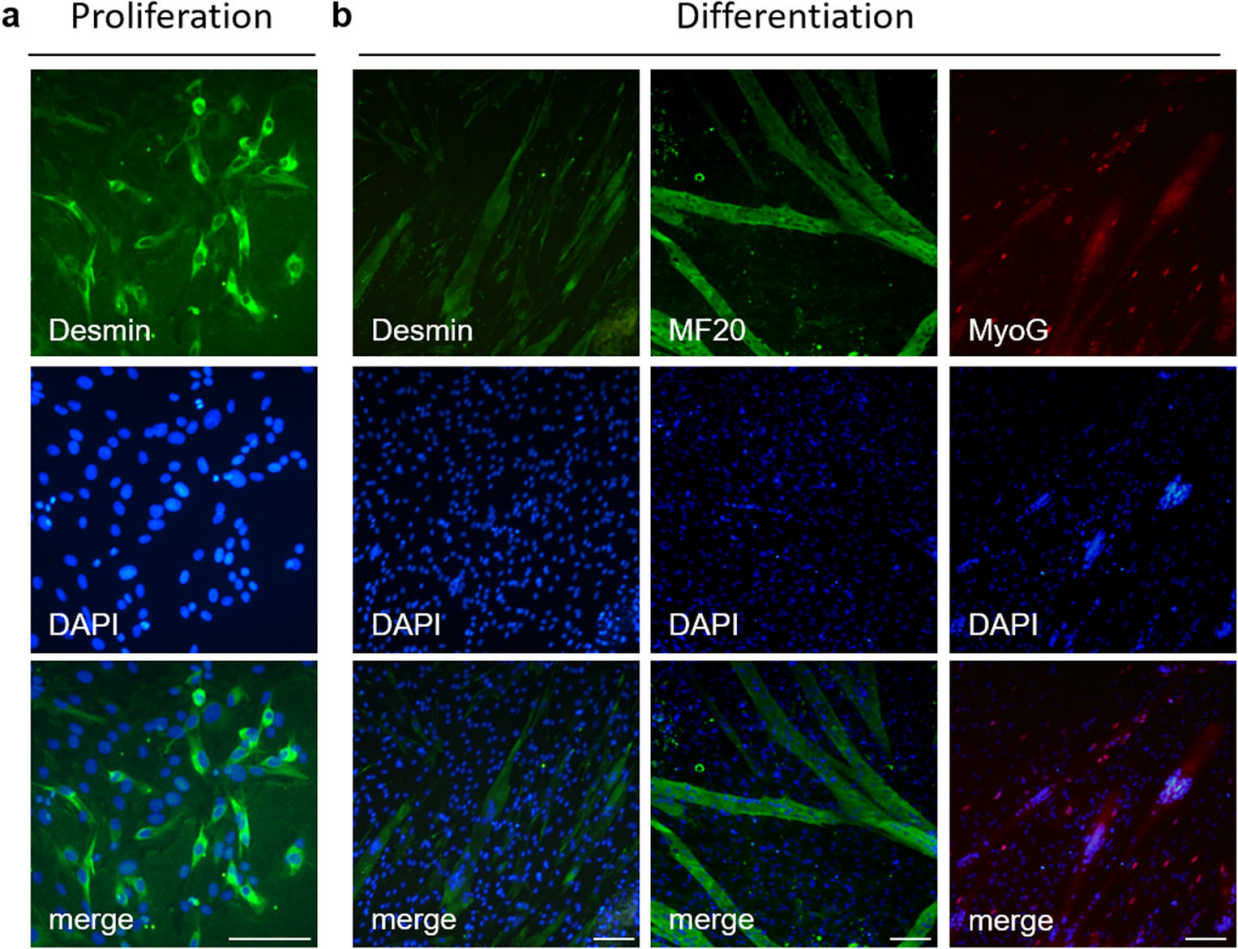
Myogenic cells from cultivated myofibers. Myofibers were seeded on collagen type I (0.5 mg/ml, Greiner Bio-one, Kremsmünster, Austria) coated cover glasses placed into a 4-well plate (Nunclon Delta surface, Thermo Fisher Scientific). After removal of myofibers after 2 d, mononuclear cells were allowed to proliferate for 6–9 d (*a*). Cells from isolated myofibers were also able to undergo myogenic differentiation and to form multinucleated myotubes (*b*). Myofibers were seeded in a Matrigel (Basement Membrane Matrix Growth Factor Reduced, BD Biosciences, Heidelberg, Germany) coated 24-well plate. Seven to 8 d after isolation (80–90% confluency), medium was changed to differentiation medium (DMEM with 4.5 g/l glucose (PAN-Biotech), 2% FBS (Thermo Fisher Scientific), 100 U/ml penicillin/streptomycin (PAN-Biotech), 2.5 μg/ml amphotericin B (PAN-Biotech)) until myotubes were formed (on average for 7 d). Cell nuclei were stained using DAPI (1 μg/ml). Leica DM4000B and the corresponding software Leica QWin V3 were used; pictures were merged using Adobe Photoshop CS5 and contrast and brightness were modified to the same degree in every sample group. *Scale bar *represents 100 μm.

Besides cultivating isolated myofibers and their associated cells in suspension culture, myofibers can be used to donate myogenic cells of high purity. The cultivation conditions can be adapted to allow migration of the cells to a surface. The coating of this surface is essential to either maintain the stem cell character (Wilschut *et al.* 2010a) or to facilitate differentiation. Twenty to 30 single myofibers/well were carefully transferred and cultured in proliferation medium (DMEM with 4.5 g/l glucose (PAN-Biotech) containing 20% fetal bovine serum (FBS, Gibco Waltham, MA), 0.5% chicken embryo extract (US Biological), 100 U/ml penicillin/streptomycin (PAN-Biotech), 2.5 μg/ml amphotericin B (PAN-Biotech)). FBS was added to the medium in order to promote cell growth and differentiation (Shefer and Yablonka-Reuveni 2005). After 2 d, before the muscle fibers adhered to the surface too tightly, they were removed by washing with DPBS. Mononuclear cells obtained from myofibers were cultured in proliferation medium until desired confluency was reached. Medium was changed every 3–4 d. Cultivation was performed in a humidified atmosphere containing 5% CO_2_ at 37°C. After proliferation or differentiation assay (Fig. 3), adherent cells were fixed with 4% PFA in PBS, washed with DPBS, and permeabilized in PBS containing either 0.1% (Desmin) or 0.5% Triton X-100 (Myosin, MyoG) for 10 or 20 min. Subsequently, cells were blocked in PBS containing 10% rabbit serum (RS/0.1% Triton X-100 (Desmin) or 20% RS/0.5% Triton X-100) for at least 1 h. For Desmin staining, cells were incubated with mouse anti-Desmin antibody (DAKO, 1:80 in PBS containing 1% RS and 0.1% Triton X-100) overnight (4°C), followed by incubation with rabbit anti-mouse IgG (H+L) secondary antibody conjugated to Alexa Fluor^®^ 488 (1:1000, Thermo Fisher Scientific) for 45 min. For Myosin and MyoG staining, cells were incubated with mouse anti-MyoG antibody (IgG1, Abcam, Berlin, Germany, 1:50) diluted in mouse anti-MHC (MF20, IgG2b, undiluted supernatant, Developmental Studies Hybridoma Bank) overnight. Antibody solution contained 0.5% Triton X-100. Both antibodies were detected by incubation with goat anti-mouse IgG1 (conjugated to Alexa Fluor^®^ 546, 1:1000, Thermo Fisher Scientific) and goat anti-mouse IgG2b antibody (conjugated to Alexa Fluor^®^ 488, 1:1000, Thermo Fisher Scientific).

Proliferating cells widely expressed the myogenic marker Desmin in our study (Fig. 3*a*). Cells which migrated from isolated myofibers were able to undergo myogenic differentiation (Fig. 3*b*). Formed myotubes expressed the filament protein Desmin as well as the late differentiation marker Myosin. Nuclei positive for the early differentiation marker Myogenin (MyoG) were found within myotubes, but also in surrounding mononucleated cells.

To the best of our knowledge, only one protocol describing the isolation of porcine myofibers exists (Wilschut *et al.* 2010b). The group put a clear focus on isolating satellite cells and side populations from the *semitendinosus* (ST) and *semimembranosus* (SM) muscle. In contrast, we established the isolation of fibers from a muscle of the lower hind limb, as it is also common in rodent studies. We used the *fibularis tertius* muscle in our study, which has accessible tendons and can be handled without touching the muscle itself, thereby preventing preparation-related damage to the muscle fibers. The method described here offers the advantage to cultivate myogenic cells on intact isolated myofibers and thereby will broaden the possibilities to study muscle cells in their endogenous niche in the pig. In future studies, our method could be adapted to other muscles and the digestion procedure might be further optimized, e.g., by using a more complex combination of enzymes in order to shorten the time needed. Co-cultivation approaches of fibers and cells, also including cell tracking via labeling dyes, are possible.

Cultivating intact myofibers ex vivo could be a bridging technology between conventional cell culture and animal experiments. Potential applications go far beyond of what is possible in rodent model systems, especially considering two major aspects: (1) improvements on muscle development and health of the pig, a worldwide important farm animal, and (2) the transferability of the results to research topics relevant for human health. Porcine-isolated myofibers provide an excellent opportunity to analyze complex signaling cascades during myogenesis and possible alterations in disease or growth retardation conditions, which cannot be addressed by conventional cell culture studies. Impaired muscle development in pigs, e.g., due to intrauterine growth retardation and/or low birth weight (Gondret *et al.* 2005; Rozance *et al.* 2018), can be better examined using this method. This might also provide a suitable model to study mechanisms and treatments for human musculoskeletal disorders, like myopathies, cachexia, or dystrophies, which approximately caused 150,000 deaths only in 2010 (Berardi *et al.* 2014). Although the method has to be further optimized, one can think of a plethora of further applications in the future, which demand for a model system originating from a large mammal: investigating (pharmacological) substances, animal feed additives, and medium supplements, toxicity tests, developing new therapeutic perspectives, assessing genetic manipulations and their implications, or studying the regenerative capacity of the muscle.
